# Theoretical Study of the NO Reduction Mechanism on Biochar Surfaces Modified by Li and Na Single Adsorption and OH Co-Adsorption

**DOI:** 10.3390/molecules29030574

**Published:** 2024-01-24

**Authors:** Qiong Su, Fang Ren, Mengmeng Lu, Jinqin Zhao, Xingchen Zhu, Tao Shen, Yan Shen, Yanbin Wang, Junxi Liang

**Affiliations:** 1 Engineering Research Center of Biomass-Functional Composite Materials of Gansu Province, College of Chemical Engineering, Northwest Minzu University, Lanzhou 730030, China; hgsq@xbmu.edu.cn (Q.S.); rfyzcsign@126.com (F.R.); 18736802474@163.com (M.L.); 18198090479@163.com (J.Z.); z244936418@163.com (X.Z.); kenan98@163.com (T.S.); 2 College of Materials Science and Engineering, Nanjing Tech University, Nanjing 211800, China; shenyan@njtech.edu.cn

**Keywords:** biochar, NO reduction, density functional theory

## Abstract

Theoretical and experimental investigations have shown that biochar, following KOH activation, enhances the efficiency of NO removal. Similarly, NaOH activation also improves NO removal efficiency, although the underlying mechanism remains unclear. In this study, zigzag configurations were employed as biochar models. Density functional theory (DFT) was utilized to examine how Li and Na single adsorption and OH co-adsorption affect the reaction pathways of NO reduction on the biochar surface. The rate constants for all reaction-determining steps (RDSs) within a temperature range of 200 to 1000 K were calculated using conventional transition state theory (TST). The results indicate a decrease in the activation energy for NO reduction reactions on biochar when activated by Li and Na adsorption, thus highlighting their beneficial role in NO reduction. Compared to the case with Na activation, Li-activated biochar exhibited superior performance in terms of the NO elimination rate. Furthermore, upon the adsorption of the OH functional group onto the Li-decorated and Na-decorated biochar models (LiOH-decorated and NaOH-decorated chars), the RDS energy barriers were higher than those of Li and Na single adsorption but easily overcome, suggesting effective NO reduction. In conclusion, Li-decorated biochar showed the highest reactivity due to its low RDS barrier and exothermic reaction on the surface.

## 1. Introduction

Nitrogen oxide (NO_x_) concentrations in the atmosphere have risen dramatically in many parts of the world over the past few decades [1,2,3]. This has harmed environmental quality, human health, and ecosystem structure and function [4]. The growing NO_x_ emissions are dominated by fossil fuel combustion, which can be reduced through combustion control and flue gas denitrification. There are two flue gas denitration technology types: dry and wet. Nonselective catalytic reduction (NSCR), selective catalytic reduction (SCR), and selective non-catalytic reduction (SNCR) are all forms of dry treatment [5]. The SCR method is currently considered the most efficient technique for NO_x_ treatment out of the three of them [6]. There are various types of SCR denitration catalysts, including noble metal, molecular sieve, and transition metal oxide catalysts. Noble metal catalysts employ costly noble metals like platinum and palladium, which exhibit poor sulfur resistance [7]. Due to their inadequate hydrothermal stability, molecular sieve catalysts are prone to pore blocking at low temperatures [8]. Although transition metal oxide catalysts have a long lifespan and a wide range of active temperatures, they do not have high denitration efficiency. Therefore, the SCR catalyst is costly and technical [9]. Of course, to comprehend the NO oxidation trends in SACs, Li et al. [10] developed a volcano model. According to this model, Fe_1_-N_4_-C is an exceptional SAC for NO oxidation. Additionally, utilizing H_2_O_2_ for NO oxidation on an Fe-N_4_-C catalyst exhibits reduced reaction energy barriers and enables the efficient deep oxidation of NO [11]. However, findings revealed that biochar effectively converted NO to N_2_ [12] while exhibiting a lower cost than SACs.

Biochar is not solely derived from burning industrial fossil fuels; recent reports have highlighted its significant contribution to char production through environmental and agricultural sources [13,14,15]. The production process involves the pyrolysis of biomass fuels, resulting in solid char formation [16]. An analysis has shown that biochar possesses suitable characteristics for various industrial applications, including its use as a catalyst or carrier [17]. Due to its innovative and effective economics and resource properties, biochar used for flue gas denitration has drawn much attention in research [18,19,20]. Previously, Shen et al. compared the denitrification activity of activated carbon and cotton biochar used as catalysts, and the results showed that biochar had a higher nitrogen oxide conversion rate [21]. Recently, Chen et al. studied Zr, Ni, and Co metal oxides doped with biochar-supported Mn oxide catalysts and found that Zr-Mn/biochar catalysts improved NO conversion and N_2_ selectivity in low-temperature selective catalytic reduction [22]. In addition, Gong et al. examined the characteristics of heterogeneous NO reduction by Chinese fir biochar, elm biochar (EB), and bamboo biochar, in which EB demonstrated the highest NO reduction rate [23]. Notably, biochar has a relatively small specific surface area, and numerous techniques have been used to increase its adsorption and catalytic capacities. Of course, chemical factors, such as oxygen functional groups and active sites, have a stronger influence on the reduction of the NO_x_ process on the surface of activated biochar than physical factors [24]. The oxygen-containing functional groups on the surface of biochar have been found to positively impact denitration efficiency [25,26]. Some researchers have also noted that, compared to non-activated char, the inclusion of alkali metals, such as Ca, Na, and K, improves denitration efficiency [27]. According to Jia et al.’s study, lotus leaf biochar’s denitrification activity was greatly enhanced by NaOH modification [28]. Similarly, after treatment with KOH, biochar derived from empty fruit bunches exhibited higher effectiveness in removing NO_x_ than untreated biochar, particularly at lower temperatures [27,29]. To sum up, on the one hand, it has been found that more functional groups containing oxygen on the surface of biochar led to greater activities and better adsorption and reduction capabilities. On the other hand, the efficiency and process of the reaction have also been demonstrated to be impacted by alkali metal adsorption [30]. Therefore, our present study would complete the theoretical analysis of the mechanism of NO reduction on the biochar surface modified by metal Na and OH co-adsorption, and it was motivated by the influence of the abovementioned research.

To obtain a better understanding of the reduction mechanisms on biochar surfaces, theoretically explored methods have often been applied [31,32,33,34,35]. Feng et al. utilized DFT to study NO reduction on biochar surfaces after KOH activation [36]. They found that K adsorbates reduce activation energy, while OH group adsorbates increase active sites. Zhang et al. discovered that adding sodium to char increases activation sites at the edge of char and that the reaction rate of NO reduction accelerates [37]. Using quantum chemical simulations, Chen et al. determined that oxygen-containing functional groups have a significant catalytic influence on flue gas denitrification [38]. Moreover, Li et al. and Zhang et al. researched the impact of adorned Na and Ca metals on the reduction mechanism of NO on the char surface, revealing that the inclusion of alkali decoration enhances the efficiency of reduction reactions [39,40]. However, the impact of decorated Li metal on NO reduction on the char surface has not yet been studied. It remains unclear how NO reduction occurs on the biochar surface following Li and OH co-adsorption. LiOH-decorated biochar was examined in this investigation, considering that Li, Na, and K belong to the same main group in the periodic table. Li and Na atoms were selected as references to explore the trend of NO reduction reactions for LiOH- and NaOH-decorated biochar. Thus, the reduction of NO on the surface of biochar was examined in the current study using an unsaturated zigzag-type edge model. DFT was used to study how Li and Na single adsorption and OH co-adsorption affected the reaction pathways of NO reduction on the biochar surface. The traditional TST was used to compute the rate constant of each reaction RDS. This work aimed to improve the understanding of the reaction mechanism of NO reduction on the surface of biochar and to provide theoretical suggestions for the NO_x_ flue gas denitrification process.

## 2. Materials and Methods

### 2.1. Models

**Figure 1:**
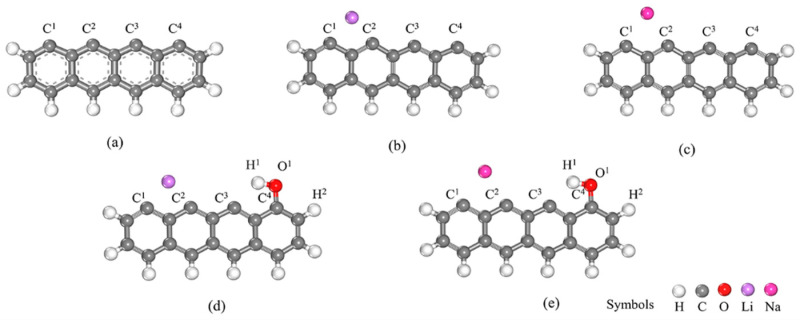
Char model used for simulating the biochar surface: (**a**) non-decorated, (**b**) Li-decorated, (**c**) Na-decorated, (**d**) decorated by Li and OH co-adsorption, and (**e**) decorated by Na and OH co-adsorption.

Previous studies have demonstrated that biomass, coal, and other solid fuels yield pyrolysis products consisting of three to seven aromatic benzene rings [41]. To simulate the mechanism of NO_x_ reduction, researchers have employed models based on these aromatic compounds, yielding precise findings [36,42]. Therefore, in this study, the zigzag model was adopted to simulate biochar (Figure 1a). The majority of metal elements in biochar exist as oxides and salts. To streamline experimental time and cost while eliminating confounding factors, single-atom decoration has been widely utilized to mimic their influence on chemical reactions [12,43]. In light of the presence of unsaturated edge atoms possessing lone pair electrons within the char model, these atoms were employed as decora sites for Li and Na atoms (Figure 1b,c). Oxygen-containing functional groups were added to the surface of biochar following treatment with LiOH and NaOH, and the identified functional groups were hydroxyl (-OH) [27]. Binding between the O atom and the biochar surface occurred [36], predominantly forming oxygen-containing functional groups at the edge sites of the char model (Figure 1d,e) [44]. Thus, the Li atoms and OH group co-adsorption and the Na atom and OH group co-adsorption represent biochar decorated by LiOH and NaOH, respectively.

### 2.2. Computational Methods

The theoretical approaches in this research are fairly similar to those in previous investigations [45,46] and were conducted using Materials Studio 8.0’s Dmol^3^ [47,48,49,50]. The exchange-correlation function employed the generalized gradient approximation (GGA-PW91). A double numerically polarized basis set (DNP) with polarization functions was used to describe the wave functions of valence electrons. All computations were performed with spin-unrestricted geometry optimization, and the all-electron basis set was utilized for solving core electron treatments. For DFT-D dispersion correction, the OBS method was adopted. The entire LST/QST procedure was implemented to obtain the reaction TS. A frequency analysis was carried out, and zero-point energy (ZPE) correction was considered for energy calculations. Relative enthalpy calculations were performed at normal atmospheric pressure and 298.15 K. The convergence requirements for structural relaxation included an energy convergence criterion of 1.0 × 10^−5^ Hartree, a force convergence criterion of 2.0 × 10^−3^ Hartree/nm, a displacement convergence criterion of 5.0 × 10^−3^ nm, and an SCF convergence criterion of 1.0 × 10^−6^ Hartree. The adsorption energy (*E*_ads_), a crucial metric for describing adsorbent capacity, was defined as follows:

(1) $$E_{\mathrm{ads}}=E_{\left(\mathrm{AB}\right)}-(E_{\left(A\right)}+E_{\left(B\right)})$$

where *E*_(AB)_ is the configuration’s energy following the optimization computation; *E*_(A)_ and *E*_(B)_ are the energies of sorbent A and adsorbate B, respectively. Based on earlier research, it was determined that adsorption falls into the chemical process when *E*_ads_ is less than −50 kJ/mol and falls into the physical absorption range when *E*_ads_ is between −30 and −10 kJ/mol.

A crucial concept in the DFT frame is the Fukui function [51], which was put forth by Parr and Yang in 1984. It is generally believed that increased reactivity corresponds with increased values of the Fukui function. Here, the finite difference approximation was used to calculate the corresponding values (Equations (2)–(5)).

(2) $$f\left(r\right)={\left\lceil \frac{\mu }{v\left(r\right)}\right\rceil }_{N}={\left\lceil \frac{\rho \left(r\right)}{N}\right\rceil }_{v\left(r\right)}$$

(3) $$f^{-}\left(r\right)=\rho_{N}-\rho_{N-1}\left(r\right)\approx \rho^{HOMO}\left(r\right)$$

(4) $$f^{+}\left(r\right)=\rho_{N+1}\left(r\right)-\rho_{N}\left(r\right)\approx \rho^{LUMO}\left(r\right)$$

(5) $$f^{0}\left(r\right)=\frac{\left[f^{+}\left(r\right)+f^{-}\left(r\right)\right]}{2}\approx \frac{\left[\rho^{HOMO}\left(r\right)+\rho^{LUMO}\left(r\right)\right]}{2}$$

*v*(*r*) is the attractive potential between the atomic nucleus and the electron at location *r*, *µ* is the chemical potential, and *N* is the electron density. In the neutral state, ionized with one lost electron, and ionized with an extra electron, the corresponding electron densities are represented by *ρ*_(_*_N_*_)_, *ρ*_(_*_N_*_+1)_, and *ρ*_(_*_N_*_−1)_.

Following Coulomb’s law [52], the electrostatic potential (ESP) at a specific location around a molecule represents the work required to transfer a unit positive charge from infinity. The precise wave function (Equation (6)) can be employed to determine this ESP.

(6) $$V_{tot}(r)=V_{nu}(r)+V_{ele}(r)=\sum_{A}\frac{Z_{A}}{|r-R_{A}|}-\int \frac{\rho (r^{\prime })}{|r-r^{\prime }|}dr^{\prime }$$

The ESP at position *r* is calculated with *V*(*r*), where *Z_A_* denotes nucleus *A*’s charge, *r* − *R_A_* represents the distance between r and nucleus *A*, and *ρ*(*r*′) refers to the electronic density at *r*′.

The TST was used in the current work to perform kinetic studies at temperatures between 300 and 1200 K. The relevant computations were based on Shermo and the TST calculator [53]:

(7) $$k=\Gamma \times \frac{k_{B}T}{\hbar }\times \frac{Q_{TS}}{Q_{A}Q_{B}}\times \exp\left(\frac{-E_{a}}{RT}\right)$$

(8) $$\Gamma =1+\left(\frac{1}{24}\right){\left(\frac{h\nu_{m}c}{k_{B}T}\right)}^{2}$$

where *ħ* is the Planck constant, *k_B_* is the Boltzmann constant, and Γ is the quantum tunneling correction factor. Accordingly, the partition functions of *TS*, reaction *A*, and product *B* are *Q_TS_*, *Q_A_*, and *Q_B_*; *R* represents the universal gas constant; the imaginary frequency of transition states, cm^−1^, is represented by *ν_m_*; and the speed of light is denoted by *c*. *E_a_* is the energy barrier for each reaction step.

## 3. Results and Discussion

### 3.1. Electronic Structure of Models

**Figure 2:**
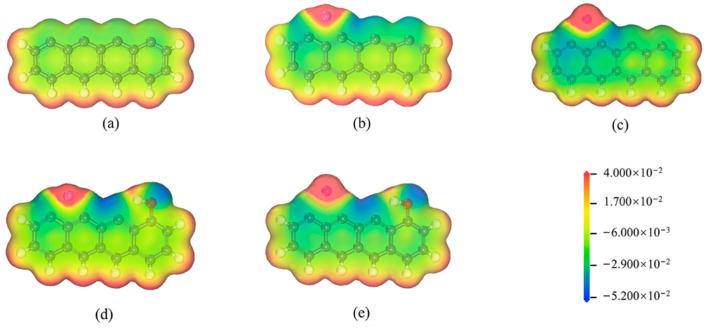
ESP of the studied biochar model: (**a**) non-decorated, (**b**) Li-decorated, (**c**) Na-decorated, (**d**) decorated by Li and OH co-adsorption, and (**e**) decorated by Na and OH co-adsorption.

Changes in the electron structure are the primary determinant of distinct surface reactivities in the biochar model. It is found that the ground-state biochar presented here exhibits a higher preference for the single-spin state over its triplet counterpart, thus establishing it as the dominant for each configuration calculation. In this paper, the impact of Li, Na, LiOH, and NaOH on the biochar surface can be evaluated by comparing the ESP before and after these treatments, as depicted in Figure 2. Hydrogen atoms engage in electron exchange with their adjacent carbon atoms, resulting in the emergence of a red region (positive charge) on hydrogen atoms and a blue region (negative charge) on carbon atoms within the carbon ring. The remaining carbon atoms (in Figure 2a) maintain a neutral charge. However, upon the introduction of a lithium atom into the carbon structure, electron transfer occurs from lithium to its neighboring carbon atoms, particularly C^2^ and C^3^. As depicted in Figure 2b, this leads to changes in the electron distribution. Thus, C^2^ and C^3^ are chosen as the optimal locations for the adsorption of NO molecules. Similarly, incorporating a Na atom into the char structure exhibits comparable behavior. The ESP of the biochar surface becomes slightly negative in the vicinity of unsaturated carbon atoms. As a result, NO tends to preferentially adsorb onto the edge atoms rather than the basal plane. Furthermore, compared to the Li-decorated char model, an apparent negative potential is shown at unsaturated C^2^ atom sites in the Na-decorated char model, but more positive potentials persist at Na atom sites (Figure 2). This finding suggests that Li atoms have more capacity to transport electrons, which increases their reactivity. The incorporation of an OH group into either the Li- or Na-decorated char model leads to the introduction of additional weak negative charges at C^2^ and C^3^ atomic sites, as well as the occupation of the active site on the char surface. The C^2^ and C^3^ atoms in the char model are therefore where NO tends to adsorb.

**Figure 3:**
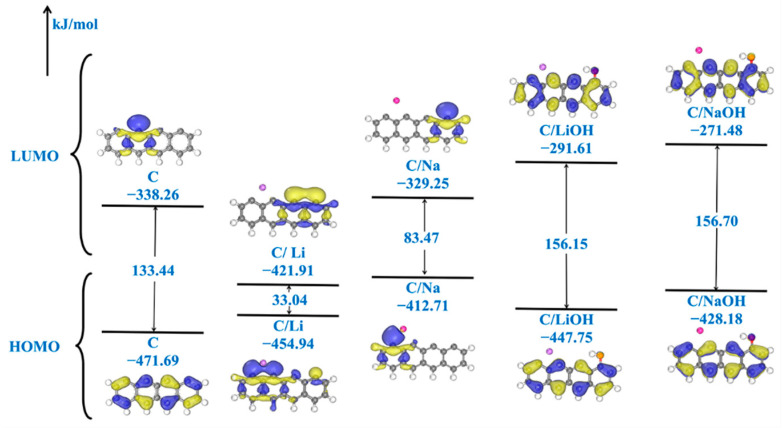
Schematic diagram of the original (C) and four modified (C/Na, C/Li, C/NaOH, and C/LiOH) char structures’ LUMO and HOMO orbitals and the corresponding energies. The yellow and blue colors indicate the phase of the orbital wave function, with blue being negative and yellow being positive.

**Figure 4:**
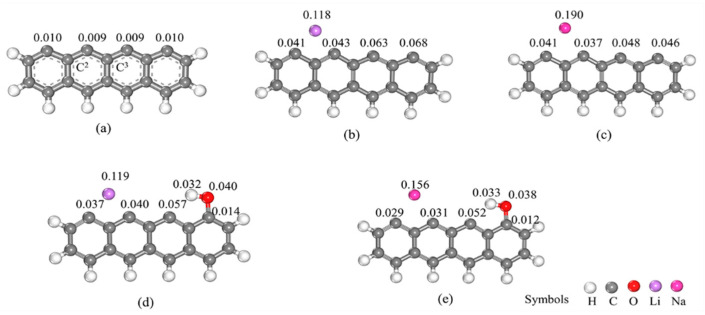
Fukui function values of the studied char model: (**a**) non-decorated, (**b**) Li-decorated, (**c**) Na-decorated, (**d**) decorated by Li and OH co-adsorption, and (**e**) decorated by Na and OH co-adsorption.

To further investigate the underlying factors contributing to the disparate impacts of Li, Na, and their hydroxides, a molecular orbital analysis is conducted on the aforementioned configurations. The highest occupied molecular orbital (HOMO) and the lowest unoccupied molecular orbital (LUMO) can serve as indicators of the chemical reactivity of a substance; the HOMO primarily acts as an electron donor, while the LUMO predominantly functions as an electron acceptor. The orbital distributions of the char decorated by Li, Na, and their hydroxides and the corresponding energy values are shown in Figure 3. The HOMO of the Li- and Na-decorated biochars, as depicted in Figure 3, localizes on the respective Na and Li atoms, as well as on the two carbon rings situated on the right side of the biochar. This indicates active sites for electrophilic reactions. Conversely, the LUMO is distributed across the benzene ring located on the right side of the biochar, suggesting its potential as an active site for nucleophilic reactions. In contrast, for the LiOH- and NaOH-decorated biochars, their HOMO disperses throughout all four carbon rings within the biochar structure due to synergistic activation between the Li/Na atoms and OH groups introduced into the biochar. It is worth mentioning that both LiOH and NaOH exhibit similar HOMO and LUMO orbitals, owing to the pi bond composition within the carbon ring structure of the biochar. According to frontier molecular orbital theory [54], the energy gap value serves as an indicator of the chemical stability of a molecule, with a larger gap value suggesting enhanced molecular stability. The HOMO, LUMO, and gap values are obtained from DFT calculations using the GGA functional, which may lead to potential underestimation. Additionally, the utilization of an incomplete basis set can introduce additional errors. Figure 3 shows significant variability in the gap values among the models, indicating the reliability of using calculated gap values for reactivity comparisons. The gap value of the Li-decorated char model is smaller than that of the Na-decorated char model, indicating that the Li-decorated char model is more active. The gap value increases when the OH functional group is adsorbed on the Li-decorated and Na-decorated char models, suggesting that the reactivity of the two structures is weakened by the addition of the OH group. In addition, the reactivity of atoms will be influenced by specific Fukui function values. As illustrated in Figure 4a, the unsaturated carbon atoms at the char edge exhibit more pronounced reactivities than the other carbon atoms within the model. This is particularly evident for C^2^ and C^3^ located at the char edge. These findings suggest that NO molecules can adsorb onto char through a side-on mode, wherein both nitrogen and oxygen atoms bind to the char model, aligning with previous studies [42,55]. When incorporating the Li atom into the char model, C^2^ and C^3^ exhibit more pronounced reactivities than other carbon atoms. This observation is consistent with the results obtained from the analysis above of the ESPs. Similarly, for the Na-decorated char model, a similar trend is observed. However, the addition of an OH group to both the Li-decorated and Na-decorated char models lowers their reactivity. As shown in Figure 3, the LiOH-decorated and NaOH-decorated chars exhibit a larger gap value, indicating decreased surface reactivity.

### 3.2. Reduction Mechanism Analysis

#### 3.2.1. Li-Decorated Biomass

**Figure 5:**
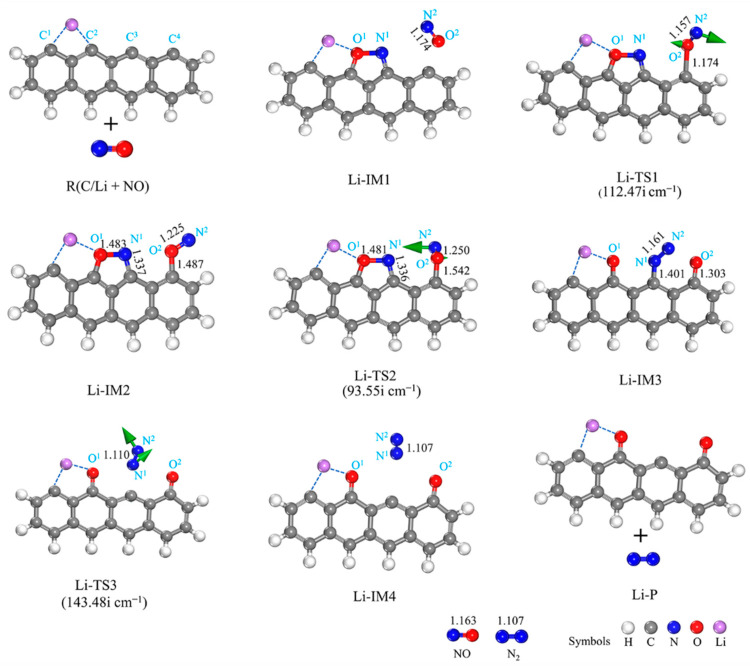
Geometrical structures of reactants, IMs, TSs, and the products of the reaction of Li-decorated char with NO. The bond lengths are given in Å. The green arrows on transition states can track the corresponding minima.

**Figure 6:**
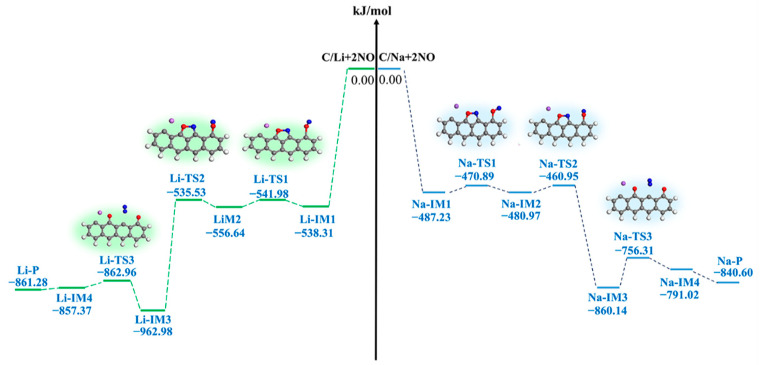
Potential energy surface for NO reactions with the Li- and Na-decorated char surfaces, respectively.

Figure 5 and Figure 6 show the structures of the intermediates (IMs) and transition states (TSs), respectively, and the potential energy surface connected to the interaction of NO with the Li- and Na-decorated chars. An analysis of Figure 5 reveals that, despite Li adsorption, there is no alteration in the reaction path compared to the unmodified char surface (Li-IM1) [36]. The presence of Li atoms on the biochar activates the C^2^ and C^3^ sites, facilitating the bidentate adsorption of NO molecules in a side-on orientation, thereby promoting chemisorption with an Eads value of −498.85 kJ/mol. Consequently, this leads to the formation of a five-membered ring structure (Li-IM1), which is a highly exothermic reaction resulting in an energy release of −538.31 kJ/mol. Subsequently, the second NO adsorbate binds to the C^4^ site through the formation of a single O^2^-C^4^ bond, which requires an energy barrier as low as 3.67 kJ/mol and leads to the formation of a stable intermediate (Li-IM2). The adsorption of two NO molecules releases a total energy of 556.64 kJ/mol. In computational studies conducted by Yang et al. [12] and Hu et al. [42], the energy released upon the adsorption of two NO molecules was found to be 546 kJ/mol and 560 kJ/mol, respectively; however, decoration with a Li atom results in a higher energy release during NO adsorption, indicating greater stability for this structure. Finally, N^2^ approaches N^1^, leading to the formation of the Li-TS2 transition state. The O^1^-N^1^ bond becomes unstable as a result of the step causing contact between the N^1^ and N^2^ atoms. The energy barrier for this reaction (from Li-IM2 to Li-IM3) is calculated as 21.11 kJ/mol. Transitioning from Li-TS2 to Li-IM3 represents an exothermic process with an energy release of −406.34 kJ/mol, resulting in the rupture of the O^1^-N^1^ bond and the disruption of the five-membered ring structure while forming a N_2_ molecule through single bonding. The energy value for Li-IM3 is determined to be −962.98 kJ/mol, which is similar to the value of −946.1 kJ/mol [40]. Subsequently, N_2_ desorbs straightforwardly from the biochar surface (from Li-IM3 to Li-IM4). As the RDS in this reaction pathway, the desorption of N_2_ exhibits an energy barrier of 100.02 kJ/mol and a reaction energy of 105.61 kJ/mol. The overall reaction energy for the complete process (C/Li + 2NO → Li-P + N_2_) amounts to −861.28 kJ/mol. Importantly, these findings reveal a reduced energy barrier in this system when compared to the original char-reduced NO reactions, thereby indicating the successful elimination of NO.

#### 3.2.2. Na-Decorated Biomass

**Figure 7:**
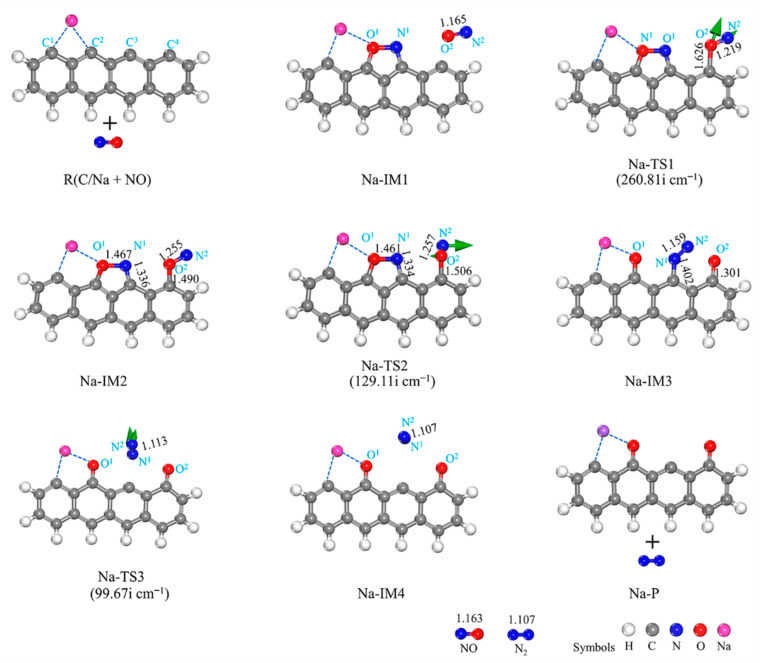
Geometrical structures of reactants, IMs, TSs, and the product of the reaction of Na-decorated char with NO. The bond lengths are given in Å. The green arrows on transition states can track the corresponding minima.

Figure 7 depicts the structures of the IMs and TSs, while Figure 6 shows the potential energy surface. An analysis of Figure 7 reveals that, despite Na adsorption, there is no alteration in the reaction pathway compared to the Li-decorated surface. Similar to the Li-decorated surface, the reaction consists of three elementary reactions. The first NO molecule exhibits bidentate binding to both the C^2^ and C^3^ sites, while the second NO molecule is monodentate-bound to the C^4^ site. Subsequently, as they approach each other, the two NO molecules undergo N^2^-O^2^ bond cleavage and form a N^1^-N^2^ bond. The third elementary reaction involves the desorption of N_2_ (from Li-IM3 to Li-IM4), which presents an energy barrier of 103.83 kJ/mol and serves as the RDS for this reaction pathway. The overall reaction energy for the complete process (C/Na + 2NO → Na-P + N_2_) is calculated as −840.60 kJ/mol. However, when the biochar interacts with Na atoms, it results in a less exothermic profile and a lower energy barrier for the NO reduction process compared to the Li-activated biochar surfaces (−840.60 vs. −861.27 kJ/mol and 103.83 vs. 100.02 kJ/mol). In conclusion, the reactivity of NO molecule reduction on the surface of the Li-decorated biochar is higher than that on the surface of the Na-decorated biochar. Throughout the entire reaction process accompanied by N_2_ desorption, various atoms, including O, Li, and Na, occupy the active sites of the biochar, leading to surface deactivation. Consequently, recycling biochar as a reactant after a reaction is not feasible.

#### 3.2.3. LiOH-Decorated Biomass

**Figure 8:**
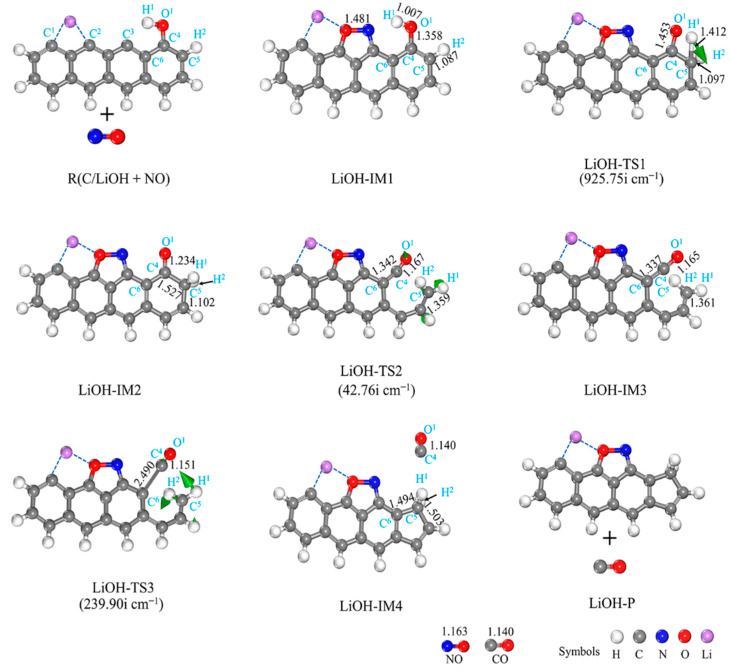
Geometrical structures of reactants, IMs, TSs, and the product of the reaction of LiOH-decorated char with NO. The bond lengths are given in Å. The green arrows on transition states can track the corresponding minima.

**Figure 9:**
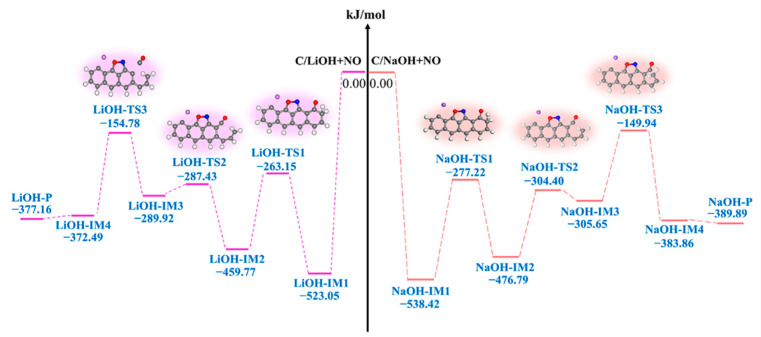
Potential energy surface for NO reactions with LiOH- and NaOH-decorated char surfaces, respectively.

Figure 8 and Figure 9 show the structures of the IMs and TSs, respectively, and the potential energy surface connected to the interaction of NO with the NaOH- and LiOH-decorated chars. The adsorption of NO onto the biochar surface sites of C^2^ and C^3^ leads to the formation of a stable intermediate, LiOH-IM1, as shown in Figure 8. Despite strong NO adsorption on the biochar surface, it does not affect the bond length of O^1^-H^1^ in the OH group or O^1^-C^4^ between the biochar surface and the OH group, with the O^1^-C^4^ and O^1^-H^1^ lengths remaining at 1.358 and 1.007 Å, respectively. Subsequently, the H^1^ atom migrates towards C^5^, with a reaction energy of 63.28 kJ/mol (LiOH-IM1 → LiOH-IM2). The transition from LiOH-IM1 to LiOH-TS1 represents the RDS of the entire reaction pathway by overcoming an energy barrier of 259.89 kJ/mol. As a result, the H^1^-O^1^ bond breaks down while forming a C^5^-H^1^ bond, thereby enhancing the O^1^ and C^4^ interactions. This results in an O^1^-C^4^ bond reduction from 1.358 Å in LiOH-IMI to 1.453 Å in LIOH-TSI, further decreasing to 1.234 Å in LiOH-IM2. Following that, the C^4^-C^5^ bond undergoes cleavage (LiOH-IM2 → LiOH-TS2 → LiOH-IM3), thereby enhancing the interaction between O^1^ and C^4^ while disrupting the six-membered carbon ring structure. The bond length between O^1^ and C^4^ experiences a significant decrease from 1.234 Å (LiOH-IM2) to a mere 0.165 Å (LiOH-IM3), resulting in a pronounced alteration. The energy barrier and reaction energy for this process are 172.34 and 169.85 kJ/mol, respectively. Next, the H^1^ atom of OH is expected to migrate towards the C^5^ site, followed by the breaking down of the C^4^-C^6^ bond and the detachment of CO molecules from the biochar surfaces, facilitating the formation of a five-membered carbon ring structure and leading to a decrease in length, as observed for the C^4^-O^1^ bonds within the CO molecules (reduced to 1.140 Å). The calculated energy barrier for this LiOH-IM3 → LiOH-TS3 → LiOH-IM4 process is determined as 135.14 kJ/mol, accompanied by a reaction energy value of −82.57 kJ/mol; thus, the overall reaction energy with this complete process totals at −377.16 kJ/mol. By comparing the impact of Li on the biochar’s reaction with NO, it becomes evident that, when considering the LiOH-decorated biochar structure, there is a significantly higher energy barrier at 261.20 kJ/mol than that observed with the Li-decorated biochar (100.02 kJ/mol). Furthermore, while reactions involving the LiOH-decorated biochar exhibit an exothermic heat release of 422.82 kJ/mol, those involving the Li-decorated biochar result in an exothermic heat release of 938.86 kJ/mol. This study demonstrates that the co-adsorption of Li and OH on biochar exhibits lower efficiency than the single adsorption of Li on char due primarily to its elevated energy barrier and lower exothermic heat release. Due to its higher RDS energy barrier in comparison, calculations regarding reduction steps involving a second NO molecule are not performed.

#### 3.2.4. NaOH-Decorated Biomass

**Figure 10:**
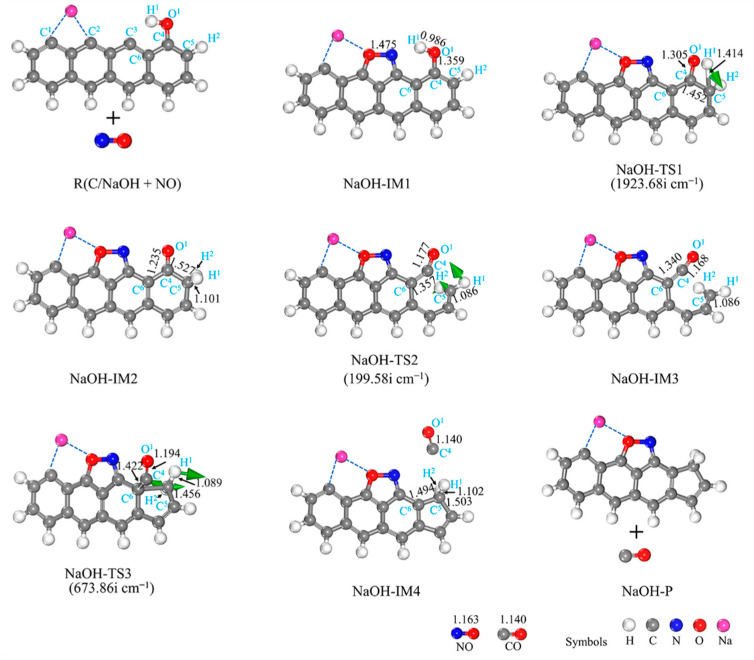
Geometrical structures of reactants, IMs, TSs, and the products of the reaction of NaOH-decorated char with NO. The bond lengths are given in Å. The green arrows on transition states can track the corresponding minima.

Figure 10 depicts the structures of IMs and TSs, while Figure 9 illustrates the potential energy surface. The presence of the NaOH-decorated char does not affect the reaction pathway, which is comparable to that of the LiOH-decorated char (Figure 10). Similar to the LiOH-decorated surface, the reaction consists of three elementary reactions starting with NO adsorption onto both C^2^ and C^3^ sites in a side-on orientation and concluding with CO desorption from the biochar surface. The reaction energy is −389.89 kJ/mol as a result (Figure 9). The chemical reaction NaOH-IM1 → NaOH-TS1 → NaOH-IM2 is the RDS for this path, with a high energy barrier of 261.20 kJ/mol. By comparing the reaction pathway of the LiOH-decorated biochar structure with NO, it can be observed that both pathways traverse an equal number of transition states. The highest energy barrier for the reaction between the NaOH-decorated biochar structure and NO is 261.20 kJ/mol, surpassing that of the LiOH-decorated biochar structure (259.89 kJ/mol). Additionally, the co-adsorption of Na and OH leads to a more exothermic process for NO reduction compared to the co-adsorption of Li and OH (−389.89 vs. −377.16 kJ/mol). Thus, on the surface of the biochar modified by the single adsorption of Li and Na, and the co-adsorption of OH groups, the reduction of NO exhibits exothermic behavior with lower energy barrier surfaces. In summary, due to its low RDS barrier and more exothermic reaction on surfaces, Li single absorption on biochar demonstrates superior reactivity compared to K atom and OH group co-absorption, as reported in the literature [36].

### 3.3. Kinetic Characteristics

**Figure 11:**
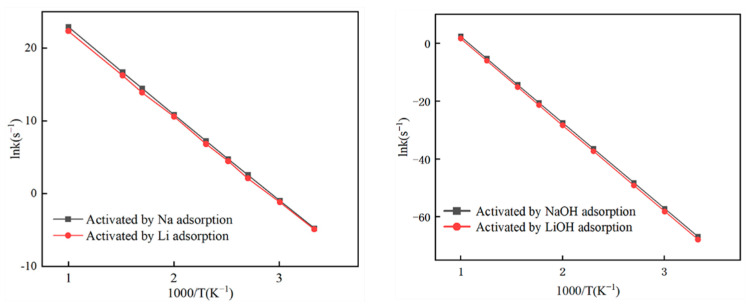
Calculated rate constants in the temperature range of 200–1200 K.

Based on the results obtained from classical TST calculations, it is inferred that N_2_ desorption from the biochar surface serves as the RDS in the reduction of NO by the Li- and Na-decorated biochars. Moreover, the adsorption of NO molecules is considered to be the RDS in the LiOH- and NaOH-decorated biochars. The temperature range of 200–1000 K was used to compute the reaction rate constants for the RDS reaction. Figure 11 illustrates the fitted linear relationships between ln(*k*) (s^−1^) and 1000/T (K^−1^). The slopes of these lines (−Δ*E*/(1000*R*)) and their intercepts (ln*A*) provide the corresponding Arrhenius parameters (−Δ*E* and *A*), as presented in Table 1. For the Li- and Na-decorated biochar surfaces, both reactions exhibit identical N_2_ desorption steps. As depicted in Figure 11 and Table 1, the reaction rate constants and activation energy for the RDS of Li/Na and LiOH/NaOH were computed, revealing a remarkable similarity between the values of Li and Na, as well as those of LiOH and NaOH. This observation can be attributed to their location in the same main group in the periodic table, thereby exhibiting analogous chemical properties. Moreover, both the Li-decorated and Na-decorated biochars follow a common mechanism for NO reduction, with identical RDSs and closely comparable energy barriers for these pivotal reactions. It is noteworthy that LiOH and NaOH exhibit equivalent reaction behavior. It is observed that the overall reaction rate exhibits an increase on the biochar surface activated by adsorbed Li and Na atoms. This is attributed to the lower energy barrier during the transition from IM2 to TS3 compared to that of the non-activated biochar surface. This investigation demonstrates that the activation energy for NO reduction on non-activated biochar is 161.5 kJ/mol, while it decreases significantly to 95.44 kJ/mol for the Li-decorated biochar and 93.80 kJ/mol for the Na-decorated biochar [36]. These results strongly imply that the NO reduction process’s activation energy is efficiently lowered by Li and Na adsorption, increasing the reaction rate. Notably, the Na-decorated biochar outperforms the Li-decorated biochar in terms of the NO reaction rate due to its lower activation energy. The RDSs in the reactions involving the LiOH- and NaOH-decorated biochars are both identified as NO molecule adsorption. When the OH group is adsorbed onto the Li-decorated and Na-decorated char models, the activation energy of NO reduction on the LiOH-decorated biochar is increased to 243.96 kJ/mol, while on the NaOH-decorated biochar, it becomes 243.91 kJ/mol; furthermore, the pre-exponential factor decreases to 1.19 × 10^13^ and 2.31 × 10^13^ s^−1^, respectively. This suggests that the presence of an OH group leads to a reduction in active sites on the surface of the biochar, thereby hindering the interaction of the LiOH- and NaOH-decorated biochars with NO. Consequently, the presence of Li and Na atoms results in a decrease in the activation energy of the reaction. In contrast, the coexistence of OH adsorbates reduces the pre-exponential factor and rate of this reaction. In summary, OH co-adsorption on the metal (Li and Na)-decorated biochar surface exhibits diminished denitration efficiency.

Based on the above data analysis, it was observed that the activation energy barrier for NO reduction on biochar surfaces activated by Li adsorption was lower and that the reaction occurred more easily compared to that of LiOH. The addition of LiOH resulted in CO molecule desorption from the biochar surface. Relevant experimental findings suggest that chars exhibit improved NO reduction at low temperatures when CO is present [56]. It is noteworthy that the denitration efficiency of biochar modified with LiOH and NaOH may be improved when considering CO reduction, although further investigation into the mechanism is required in subsequent phases.

## 4. Conclusions

The reduction mechanism of NO on modified biochar surfaces was investigated in this study using DFT and classical TST methods. Specifically, our focus was on examining the impact of the single adsorption of Li and Na atoms, as well as co-adsorption with OH, on the reaction process. (1) Based on our findings, it could be concluded that, while the adsorption of Li and Na atoms did not significantly alter the reaction mechanism, it resulted in a notable decrease in the activation energy by 66.06 and 67.70 kJ/mol, respectively. These results suggest that activating biochar surfaces through Li and Na adsorption offers significant advantages for enhancing the efficiency of NO reduction reactions. (2) Compared to the Li-decorated biochar surface, adsorption with Na atoms led to a less exothermic and higher energy barrier for the NO reduction process. Therefore, the Li-decorated biochar exhibited a superior rate of NO elimination. The Li-decorated biochar demonstrated a higher reaction rate than the Na-decorated biochar, with an activation energy of 93.80 kJ/mol. (3) The utilization of LiOH and NaOH species for biochar activation resulted in a complete alteration of the reaction mechanism, leading to benzene ring cleavage and CO liberation from the surface. Consequently, there was a reduction in the overall reaction rate, accompanied by an increase in the activation energy. Notably, adsorption by OH groups decreased the number of active sites on the biochar surface. (4) This study found that the LiOH-decorated and NaOH-decorated biochars exhibited RDS energy barriers of 259.89 kJ/mol and 261.20 kJ/mol, respectively. The RDS energy barriers were found to have been easily crossed, indicating an efficient reduction of NO emissions. (5) The highest energy barriers and reaction rates among the four reaction paths could be ranked in order of increasing difficulty as follows: Li-decorated biochar, Na-decorated biochar, LiOH-decorated biochar, and NaOH-decorated biochar. Therefore, Li-decorated biochar demonstrates superior performance in terms of the NO elimination rate.

## Figures and Tables

**Table 1:** Fitted kinetic parameters of the Arrhenius expression.

	Li-Decorated	Na-Decorated	LiOH-Decorated	NaOH-Decorated
Activation energy (kJ/mol)	95.44	93.80	243.96	243.51
Pre-exponential factor A (s^−1^)	2.70 × 10^14^	1.80 × 10^14^	1.19 × 10^13^	2.31 × 10^13^

## Data Availability

The data presented in this study are available on request from the corresponding author. The data are not publicly available due to data sharing does not apply to this article as no new data were analyzed in this study.

## References

[1] Galloway J.N., Dentener F.J., Capone D.G., Boyer E.W., Howarth R.W., Seitzinger S.P., Asner G.P., Cleveland C.C., Green P.A., Holland E.A. (2004). Nitrogen cycles: Past, present, and future. Biogeochemistry.

[2] Sutton M.A., Oenema O., Erisman J.W., Leip A., van Grinsven H., Winiwarter W. (2011). Too much of a good thing. Nature.

[3] Song W., Liu X.-Y., Hu C.-C., Chen G.-Y., Liu X.-J., Walters W.W., Michalski G., Liu C.-Q. (2021). Important contributions of non-fossil fuel nitrogen oxides emissions. Nat. Commun..

[4] Clark C.M., Tilman D. (2008). Loss of plant species after chronic low-level nitrogen deposition to prairie grasslands. Nature.

[5] Wei Y., Li D., Qiao J., Guo X. (2023). Recovery of spent SCR denitration catalyst: A review and recent advances. J. Environ. Chem. Eng..

[6] Hao S., Yuling L., Yang J. (2022). Synthesis of Cu-BTC by room temperature hydrothermal and its low temperature SCR denitration. J. Mol. Struct..

[7] Shi J., Zhang Y., Zhu Y., Chen M., Zhang Z., Shangguan W. (2019). Efficient Fe-ZSM-5 catalyst with wide active temperature window for NH_3_ selective catalytic reduction of NO: Synergistic effect of isolated Fe^3+^ and Fe_2_O_3_. J. Catal..

[8] Zhang Z., Li J., Tian J., Zhong Y., Zou Z., Dong R., Gao S., Xu W., Tan D. (2022). The effects of Mn-based catalysts on the selective catalytic reduction of NOx with NH3 at low temperature: A review. Fuel Process. Technol..

[9] Van Caneghem J., De Greef J., Block C., Vandecasteele C. (2016). NO_x_ reduction in waste incinerators by selective catalytic reduction (SCR) instead of selective non-catalytic reduction (SNCR) compared from a life cycle perspective: A case study. J. Clean. Prod..

[10] Yang W., Feng Y., Chen X., Wu C., Wang F., Gao Z., Liu Y., Ding X., Li H. (2022). Understanding Trends in the NO Oxidation Activity of Single-Atom Catalysts. J. Environ. Chem. Eng..

[11] Yang W., Chen L., Zhou B., Jia Z., Liu X., Liu Y., Li H., Gao Z. (2023). NO Oxidation Using H_2_O_2_ at a Single-Atom Iron Catalyst. J. Phys. Chem. C.

[12] Yang M., Liu C., Xu L., Dong M., Wang Z., Shen B., Kong W., Wang X. (2023). Catalytic mechanism of bi-alkali-metal-doped char in heterogeneous reduction of NO: A density functional theory study. Energy.

[13] Wei J.T., Wang M., Tang G.J., Akhtar M.A., Xu D.L., Song X.D., Yu G.S., Li B., Zhang H., Zhang S. (2022). Advances on in-situ analysis of char structure evolution during thermochemical conversion of coal/biomass: A review. Fuel Process. Technol..

[14] Kazemi Shariat Panahi H., Dehhaghi M., Ok Y.S., Nizami A.-S., Khoshnevisan B., Mussatto S.I., Aghbashlo M., Tabatabaei M., Lam S.S. (2020). A comprehensive review of engineered biochar: Production, characteristics, and environmental applications. Clean. Prod..

[15] Devaraja U.M.A., Dissanayake C.L.W., Gunarathne D.S., Chen W.H. (2022). Oxidative torrefaction and torrefaction-based biorefining of biomass: A critical review. Biofuel Res. J..

[16] Foong S.Y., Liew R.K., Yang Y., Cheng Y.W., Yek P.N.Y., Wan Mahari W.A., Lee X.Y., Han C.S., Vo D.-V.N., Van Le Q. (2020). Valorization of biomass waste to engineered activated biochar by microwave pyrolysis: Progress, challenges, and future directions. Chem. Eng. J..

[17] Mishra R.K., Mohanty K. (2022). Pyrolysis of low-value waste sawdust over low-cost catalysts: Physicochemical characterization of pyrolytic oil and value-added biochar. Biofuel Res. J..

[18] Deng W., Tao C., Cobb K., Zhou H., Su Y., Ruan R. (2020). Catalytic oxidation of NO at ambient temperature over the chars from pyrolysis of sewage sludge. Chemosphere.

[19] Li N., Wang Y., Cui S., Jin X. (2020). Experimental and Kinetic Investigation on NO Reduction by Rice Husk Char and Catalytically with CO. Appl. Sci..

[20] Xu J., Zhang X., Sun Y., Long H., Zheng Z. (2020). Improvement of low-temperature NH3-SCR catalytic activity over Mn-Ce oxide catalysts supported on sewage sludge char activated with KOH and H_3_PO_4_. Korean J. Chem. Eng..

[21] Shen B., Chen J., Yue S., Li G. (2015). A comparative study of modified cotton biochar and activated carbon based catalysts in low temperature SCR. Fuel.

[22] Chen L., Ren S., Liu W., Yang J., Chen Z., Wang M., Liu Q. (2021). Low-temperature NH_3_-SCR activity of M (M = Zr, Ni and Co) doped MnO supported biochar catalysts. J. Environ. Chem. Eng..

[23] Gong Q.-C., He L.-Q., Zhang L.-H., Duan F. (2021). Comparison of the NO heterogeneous reduction characteristics using biochars derived from three biomass with different lignin types. J. Environ. Chem. Eng..

[24] Jo Y.B., Cha J.S., Ko J.H., Shin M.C., Park S.H., Jeon J.-K., Kim S.-S., Park Y.-K. (2011). NH_3_ selective catalytic reduction (SCR) of nitrogen oxides (NO_x_) over activated sewage sludge char. Korean J. Chem. Eng..

[25] Wang Y., Qin N., Cui S., Ma X., Peng S. (2020). Influence of Biochar Composition and Micro-Structure on the Denitration of Flue Gases at High Temperature. Appl. Sci..

[26] Li Q., Hou Y., Wang J., Liu Y., Xiang N., Huang Z. (2020). Superiority of Raw Biomass and Potassium Hydroxide in Preparation of Ultrahigh Nitrogen Doping of Carbon for NH_3_-SCR Reaction. ACS Sustain. Chem. Eng..

[27] Chen H., Chen D., Hong L., Dou X. (2015). Recycle Sewage Sludge Char as Flue Gas De-NO_x_ Catalyst within Low Temperature Ranges. Energy Procedia.

[28] Jia X., Peng R., Huang H., Dan J., Lu M., Zhang D., Wang J., Li D., Fang H., Yu C. (2022). Lotus leaves-derived MnO_x_/biochar as an efficient catalyst for low-temperature NH_3_-SCR removal of NO_x_: Effects of modification methods of biochar. J. Chem. Technol. Biotechnol..

[29] Jongha L., Hoyeon L., Seungryong C. (2018). Sinogram synthesis using convolutional-neural-network for sparsely view-sampled CT. Proc. SPIE.

[30] Chen H., Chen D., Hong L. (2015). Influences of activation agent impregnated sewage sludge pyrolysis on emission characteristics of volatile combustion and De-NO_x_ performance of activated char. Appl. Energy.

[31] Gao Z., Li M., Sun Y., Yang W. (2018). Effects of oxygen functional complexes on arsenic adsorption over carbonaceous surface. J. Hazard. Mater..

[32] Gao Z., Zhao M., Yan G., Huang H., Yang W., Ding X., Wu C., Gates l.D. (2020). Identifying the active sites of carbonaceous surface for the adsorption of gaseous arsenic trioxide: A theoretical study. Chem. Eng. J..

[33] Yan G., Gao Z., Zhao M., Yang W., Ding X. (2020). A comprehensive exploration of mercury adsorption sites on the carbonaceous surface: A DFT study. Fuel.

[34] Zhang H., Song H., Yuan R., Zhang X., Yu H., Zhao Y., Jiang T. (2018). Polyene phosphatidylcholine overcomes oxaliplatin resistance in human gastric cancer BGC823 cells. Biochem. Biophys. Res. Commun..

[35] Yang W., Gao Z., Liu X., Ma C., Ding X., Yan W. (2019). Directly catalytic reduction of NO without NH_3_ by single atom iron catalyst: A DFT calculation. Fuel.

[36] Feng K., Hu Y., Cao T. (2022). Mechanism of Fuel Gas Denitration on the KOH-Activated Biochar Surface. J. Phys. Chem. A.

[37] Zhang X.-X., LÜ X.-X., Wu H.-X., Xie M., Lin R.-Y., Zhou Z.-J. (2020). Microscopic mechanism for effect of sodium on NO heterogeneous reduction by char. J. Fuel Chem. Technol..

[38] Chen P., Gu M., Chen G., Liu F., Lin Y. (2019). DFT study on the reaction mechanism of N_2_O reduction with CO catalyzed by char. Fuel.

[39] Li Y., Niu S.-L., Wang Y.-Z., Han K.-H., Zhou W.-B., Wang J. (2021). Mechanism of N_2_O reduction by biomass gasification gas reburning. J. Fuel Chem. Technol..

[40] Zhang X., Xie M., Wu H., Lv X., Zhou Z. (2020). DFT study of the effect of Ca on NO heterogeneous reduction by char. Fuel.

[41] Perry S.T., Hambly E.M., Fletcher T.H., Solum M.S., Pugmire R.J. (2000). Solid-state ^13^C NMR characterization of matched tars and chars from rapid coal devolatilization. Proc. Combust. Inst..

[42] Feng K., Hu Y., Cao T. (2022). Effect of K-decoration on the generation and reduction of N_2_O onto a biochar surface. Fuel.

[43] Liu J., Xia Y.-g., Sun H.-d., Hu B., Zhang B., Lu Q. (2024). Insight into the fate of nitrogen during char thermal conversion and the influence mechanism of potassium: A theoretical research. Sci. Total Environ..

[44] Chen P., Gu M., Chen X., Chen J. (2019). Study of the reaction mechanism of oxygen to heterogeneous reduction of NO by char. Fuel.

[45] Yang J., Yuan S., Wang S., Yang M., Shen B., Zhang Q., Zhang Z., Wang F., Xu L., Wang Z. (2020). Density Functional Theory Study on the Effect of Sodium on the Adsorption of NO on a Char Surface. Energy Fuels.

[46] Yang J., Chen L., Su J., Huang Y., Zhang M., Gao M., Yang M., Yuan S., Wang X., Shen B. (2022). Mechanism on the effect of sodium on the heterogeneous reduction reaction of NO by Char(N). Fuel.

[47] Liu X.D., Wei Q., Huang W.B., Zhou Y.S., Zhang P.F., Xu Z.S. (2020). DFT insights into the stacking effects on HDS of 4,6-DMDBT on Ni-Mo-S corner sites. Fuel.

[48] Liu X.D., Ding S.J., Wei Q., Zhou Y.S., Zhang P.F., Xu Z.S. (2021). DFT insights in to the hydrodenitrogenation behavior differences between indole and quinoline. Fuel.

[49] Wu C.C., Yang W.J., Wang J.Y., Kannaiyan R., Gates I.D. (2021). CO_2_ adsorption and dissociation on single and double iron atomic molybdenum disulfide catalysts: A DFT study. Fuel.

[50] Accelrys Inc. (2014). Malerials Studio Version 8.0.

[51] Parr R.G., Yang W.T. (1984). Density functional-approach to the frontier-electron theory of chemical-reactivity. J. Am. Chem. Soc..

[52] Murray J.S., Politzer P. (2011). The electrostatic potential: An overview. Wires Comput. Mol. Sci..

[53] Lu T., Chen Q.X. (2021). Shermo: A general code for calculating molecular thermochemistry properties. Comput. Theor. Chem..

[54] Ruiz-Morales Y. (2002). HOMO-LUMO gap as an index of molecular size and structure for polycyclic aromatic hydrocarbons (PAHs) and asphaltenes: A theoretical study. I. J. Phys. Chem. A.

[55] Liu J., Xia Y.-G., Wu Y.-W., Zhou X.-Y., Hu B., Lu Q. (2023). Microscopic mechanism for the effect of potassium on heterogeneous NO–char(N) interaction: A theoretical account. Fuel Process. Technol..

[56] Ke X., Jiang L., Li Y., Yang H., Lyu J., Zhang M. (2022). A comprehensive study of char reaction kinetics: Does CO always promote NO reduction on char surface?. Fuel.

